# Supplementary material for: Modelling Skylarks (Alauda arvensis) to Predict Impacts of Changes in Land Management and Policy: Development and Testing of an Agent-Based Model
Source: PLoS One. 2013 Jun 6;8(6):e65803. doi: 10.1371/journal.pone.0065803 (PMC3675089; doi:10.1371/journal.pone.0065803)
Supplement: Supporting Information S4 — The skylark ODdox as a zipped archive. (ZIP) [file pone.0065803.s004.zip › Skylark_ODdox/class_rodenticide_manager-members.html]

ALMaSS Skylark ODdox: Member List


ALMaSS Skylark ODdox
 2.0


- Main Page
- Related Pages
- Classes
- Files

- Class List
- Class Index
- Class Hierarchy
- Class Members

RodenticideManager Member List

This is the complete list of members for RodenticideManager, including all inherited members.

|  |  |  |
| --- | --- | --- |
| Diffuse(int a\_x, int a\_y) | RodenticideManager | inline |
| DistributeRing(int a\_x, int a\_y, double a\_amount) | RodenticideManager | inline |
| DoDeath(void) | RodenticideManager | inline |
| DoDiffuse(void) | RodenticideManager | inline |
| DoImmigration(void) | RodenticideManager |  |
| DoPlaceBait() | RodenticideManager |  |
| DumpRodenticideMap(string a\_fname) | RodenticideManager |  |
| GetBaitStartDate(TTypesBaitLocation) | RodenticideManager |  |
| GetMap(int a\_x, int a\_y) | RodenticideManager | inline |
| GetMapTwin(int a\_x, int a\_y) | RodenticideManager | inline |
| GetRodenticide(unsigned a\_x, int a\_y) | RodenticideManager | inline |
| InitiateRodenticidePolyData(string a\_fname) | RodenticideManager |  |
| m\_BaitList | RodenticideManager | protected |
| m\_deathRate | RodenticideManager | protected |
| m\_diffusionRate | RodenticideManager | protected |
| m\_gridsize | RodenticideManager | protected |
| m\_height | RodenticideManager | protected |
| m\_heightm1 | RodenticideManager | protected |
| m\_immigrationRate | RodenticideManager | protected |
| m\_land | RodenticideManager | protected |
| m\_pmouse\_map\_main | RodenticideManager | protected |
| m\_pmouse\_map\_twin | RodenticideManager | protected |
| m\_seasonstartprob | RodenticideManager | protected |
| m\_width | RodenticideManager | protected |
| RecordRodenticidePolyData(string a\_fname) | RodenticideManager |  |
| RodenticideManager(string fname, Landscape \*a\_land) | RodenticideManager |  |
| SetMap(int a\_x, int a\_y, double a\_p) | RodenticideManager | inline |
| SetMapTwin(int a\_x, int a\_y, double a\_p) | RodenticideManager | inline |
| SetMapTwinMult(int a\_x, int a\_y, double a\_p) | RodenticideManager | inline |
| SetMapTwinP(int a\_x, int a\_y, double a\_p) | RodenticideManager | inline |
| ShouldPlaceBait(TTypesBaitLocation) | RodenticideManager |  |
| Tick(void) | RodenticideManager |  |
| ~RodenticideManager(void) | RodenticideManager | virtual |


- Generated on Thu Jan 10 2013 13:15:37 for ALMaSS Skylark ODdox by
   1.8.1.1
